# A fluorescent curcumin-based Zn(II)-complex reactivates mutant (R175H and R273H) p53 in cancer cells

**DOI:** 10.1186/1756-9966-32-72

**Published:** 2013-10-07

**Authors:** Alessia Garufi, Daniela Trisciuoglio, Manuela Porru, Carlo Leonetti, Antonella Stoppacciaro, Valerio D’Orazi, Maria Laura Avantaggiati, Alessandra Crispini, Daniela Pucci, Gabriella D’Orazi

**Affiliations:** 1 Department of Experimental Oncology, Regina Elena National Cancer Institute, 00159 Rome, Italy; 2 Experimental Medicine and Pathology Department, II Faculty S. Andrea, 00198 Rome, Italy; 3 Department of Surgical Sciences, Sapienza University, 00100 Rome, Italy; 4 Department of Oncology, Lombardi Comprehensive Cancer Center, Georgetown University, Washington, DC, USA; 5 Centro di Eccellenza CEMIF.CAL-LASCAMM, CR-INSTM, Department of Chimica e Tecnologie Chimiche, University of Calabria, 87100 Cosenza, Italy; 6 Department of Medical, Oral and Biotechnological Sciences, University “G. d’Annunzio”, 66013 Chieti, Italy

**Keywords:** Mutant p53, Protein conformation, p53 transcriptional activity, DNA binding, Zinc complex, Cancer therapy, Glioblastoma, Gene expression

## Abstract

**Background:**

Mutations of the p53 oncosuppressor gene are amongst the most frequent aberration seen in human cancer. Some mutant (mt) p53 proteins are prone to loss of Zn(II) ion that is bound to the wild-type (wt) core, promoting protein aggregation and therefore unfolding. Misfolded p53 protein conformation impairs wtp53-DNA binding and transactivation activities, favouring tumor growth and resistance to antitumor therapies. Screening studies, devoted to identify small molecules that reactivate mtp53, represent therefore an attractive anti-cancer therapeutic strategy. Here we tested a novel fluorescent curcumin-based Zn(II)-complex (Zn-curc) to evaluate its effect on mtp53 reactivation in cancer cells.

**Methods:**

P53 protein conformation was examined after Zn-curc treatment by immunoprecipitation and immunofluorescence assays, using conformation-specific antibodies. The mtp53 reactivation was evaluated by chromatin-immunoprecipitation (ChIP) and semi-quantitative RT-PCR analyses of wild-type p53 target genes. The intratumoral Zn-curc localization was evaluated by immunofluorescence analysis of glioblastoma tissues of an ortothopic mice model.

**Results:**

The Zn-curc complex induced conformational change in p53-R175H and -R273H mutant proteins, two of the most common p53 mutations. Zn-curc treatment restored wtp53-DNA binding and transactivation functions and induced apoptotic cell death. In vivo studies showed that the Zn-curc complex reached glioblastoma tissues of an ortothopic mice model, highlighting its ability to crossed the blood-tumor barrier.

**Conclusions:**

Our results demonstrate that Zn-curc complex may reactivate specific mtp53 proteins and that may cross the blood-tumor barrier, becoming a promising compound for the development of drugs to halt tumor growth.

## Introduction

The p53 oncosuppressor is a transcription factor whose activation in response to DNA damage leads to cell cycle arrest, senescence, or apoptosis [1]. Approximately 55% of human tumors have genetically identifiable loss of p53 function mainly by point mutation in the core (DNA-binding) domain (DBD) [2], http://p53.iarc.fr. The DBD (residues 94–312) binds the single Zinc(II) ion and p53, as many transcription factors, uses zinc to maintain structure and transactivation function for its wild-type (wt) activity [3]. Mutant p53 (mtp53) proteins are prone to loss of Zn(II) ion, which consequently promotes aggregation and therefore protein misfolding [4]. The major consequence of core mutation is loss of sequence-specific DNA binding to the canonical wtp53-binding site of target genes with loss of p53 oncosuppressor function. In some cases though, mtp53 proteins may acquire pro-oncogenic functions contributing to tumor progression [5]; moreover, loss of the ability of mtp53 to induce the expression of the E3-ubiquitin ligase MDM2 is thought to be responsible for the mtp53 enhanced stability [6]. These observations, and the finding that mtp53 protein is often expressed at high levels in tumors, make mtp53 reactivation an attractive strategy as anticancer therapy [7]. Many screening studies are underway to identify small molecules that reactivate mtp53 by acting on the equilibrium of native and denatured protein immediately after translation, by acting on the misfolded states, or by alleviating the mtp53 pro-oncogenic affects (i.e., mutp53/p73 interaction) [5,7,8].

In previous studies we found that ZnCl_2_ treatment induced the transition of mutant p53 protein into a functional conformation [9-12]. Although we found that ZnCl_2_ treatment did not induce cell death by itself, it restored mt-p53-carrying cell sensitivity to chemotherapy allowing tumor regression [9-12]. Here we aimed at examine the effect of a novel Zinc compound, a heteroleptic pentacoordinated (bpy-9)Zn(curc, Cl) complex (hereafter indicated as Zn-curc) containing a 4,4’-disubstituted-2,2′-bipyridine as main ligand and curcumin (curc) and chloride (Cl) as ancillary ligands [13,14], in mutant p53-carrying cancer cells. The presence of the curcumin framework in the Zn-curc complex allows intrinsic fluorescence activity, therefore we attempted to exploit this feature to evaluate the intratumoral distribution of Zn-curc in an ortothopic model of glioblastoma in mice. We choose to use glioblastoma because it is the most common and lethal primary central nervous system (CNS) where inactivation of the p53 gene and the presence of aberrant p53 expression are often reported [15]. Moreover, glioblastoma presents unique challenges to therapy due to its location, aggressive biological behaviour, angiogenesis and diffuse infiltrative growth. Thus, glioblastoma becomes easily chemoresistant, besides, the existence of blood-tumor barrier (BTB) represents an obstacle influencing the therapeutic efficacies via systemic administration [16].

In this study, we analyzed the biological effect of the novel Zn-curc complex in several cancer cell lines carrying different p53 mutations. Immunoprecipitation studies with conformation-specific antibodies were performed to evaluate p53 protein conformation after treatment. Finally, immunofluorescence analysis of glioblastoma tissues, of an ortothopic mice model treated with Zn-curc, was performed lo look for Zn-curc localization. Our results show that Zn-curc was effective in reactivating at least two of the most common p53 mutations, such as R175H and R273H, and to directly induce apoptotic cell death. Moreover, Zn-curc localized inside glioblastoma tissues suggesting its ability to cross the blood-tumor barrier.

## Materials and methods

### Ethics statement

All animals were handled in strict accordance with good animal practice as defined by the relevant national and/or local animal welfare bodies, and in accordance with the Italian and European legislation. All work was performed in accordance with the guidelines of the National Cancer Institute Regina Elena, where there is currently no active Ethical Committee for animal research, and has been filed with the Veterinary Service Unit and the Italian Ministry of Health, in accordance with the Italian and European legislation.

### Cell culture and treatments

The human colon cancer RKO (wtp53), glioblastoma U373MG (expressing R273H p53 mutation) and T98G (expressing M237I p53 mutation) cell lines were maintained in RPMI-1640 (Life Technology-Invitrogen), while human SKBR3 (expressing R175H p53 mutation), MD-MBA231 (expressing p53 mutation R280K) breast cancer cell lines and human fibroblasts (HF) (kindly provided by S. Soddu, Regina Elena National Cancer Institute, Rome, Italy) were maintained in DMEM (Life Technology-Invitrogen), all supplemented with 10% heat-inactivated fetal bovine serum plus glutamine and antibiotics.

The following reagents were used: a heteroleptic pentacoordinated (bpy-9)Zn(curc, Cl) complex containing a 4,4′-disubstituted-2,2′-bipyridine as main ligand and curcumin (curc) and chloride (Cl) as ancillary ligands [13] was dissolved in DMSO and used at the indicated concentrations; curcumin was prepared as previously reported [17]; pifithryn-α (PFT-α) (ENZO Life Sciences, Lausen Switzerland) was dissolved in DMSO and used at 30 μM; adryamycin (ADR) was used at 2 μg/ml and ZnCl_2_ was used at 100 μM.

### Viability and colony assays

Subconfluent cells were plated in triplicate in 60 mm Petri dishes and 24 h later treated with Zn-curc complex (20-50-100 μM) for 24 and 48 h. Both floating and adherent cells were collected and cell viability was determined by Trypan blue exclusion by direct counting with a haemocytometer, as reported. The percentage of cell viability, as blue/total cells, was assayed by scoring 200 cells per well three times.

For long-term cell survival, subconfluent cells were plated in 60 mm Petri dishes and 24 h later treated with Zn-curc complex (20-50-100 μM). Twenty-four hours later, plates were washed with PBS and fresh medium was added. Death-resistant colonies were stained with crystal violet 14 days later.

### Cell death/PI staining

Cell death was detected by cytofluorimetric analysis of propidium iodide (PI)-stained cells staining. Briefly, cells floating were collected by centrifugation and pooled with adherent cells recovered from the plates, fixed in 80% ethanol and stained in a PBS solution containing PI (62.5 mg/mL; Sigma-Aldrich), and RNase A (1.125 mg/mL; Sigma-Aldrich). Samples were acquired with a FACScan instrument (Becton Dickinson) and the percentage of cells in sub-G1 compartment was calculated using ModFit LT software (Becton Dickinson).

### Western blotting and p53 conformational immunoprecipitation

Total cell extracts were prepared by incubation in lysis buffer (50 mM Tris–HCl, pH 7.5, 150 mM NaCl, 5 mM EDTA, 150 mM KCl, 1 mM dithiothreitol, 1% Nonidet P-40) and a mix of protease inhibitors and resolved by 9-12% SDS-polyacrilamide gel electrophoresis. Proteins were transferred to a polyvinylidene difluoride membrane (PVDF, Millipore) and membranes were blocked with 5% nonfat dry milk in PBS and incubated with the primary antibodies followed by an anti-immunoglobulin–G-horseradish peroxidase antibody (BioRad). Immunoblotting was performed with the following antibodies: monoclonal anti-poly(ADP-ribose) polymerase (PARP, BD Pharmingen, CA, USA), monoclonal anti-p53 (Ab-DO1), polyclonal anti-p53 (FL393) and polyclonal anti-Bax (all from Santa Cruz Biotechnology), purified mouse anti-phospho-Histone H2AX (Ser139) (Millipore, clone JBW301; kindly provided by S. Soddu, Regina Elena National cancer Institute, Rome, Italy) and monoclonal anti-β-actin (Calbiochem). Enzymatic signals were visualized by chemoluminescence (ECL kit, Amersham Corporation).

P53 protein conformation was evaluated essentially as described [9]. Briefly, cells were lysed in immunoprecipitation buffer (10 mM Tris, pH 7.6; 140 mM NaCl; 0.5% NP40, and protease inhibitors) for 20 min on ice, and cleared by centrifugation. Pre-cleared supernatants (200 μg) were immunoprecipitated overnight at 4°C with the conformation-specific monoclonal antibodies Pab1620 (wild-type specific) and PAb240 (mutant specific) (Calbiochem) [18,19] pre-adsorbed to protein G-agarose (Pierce). Immunocomplexes were collected by centrifugation, separated by 9% SDS-PAGE and blotted onto PVDF membrane (Millipore). Immunoblotting was performed with rabbit polyclonal anti-p53 (FL393).

### Immunofluorescence staining

The cells were grown on coverslips and treated with Zn-curc (100 μM) for 24 h. After treatment, cells were fixed in 4% formaldehyde for 10 min and then premeabilized with 0.5% Triton X-100 for 5 min before staining with conformation antibodies PAb1620 and PAb240 at 1:200 dilution in PBST, overnight at 4°. Cells were then visualized on a Nikon Eclipse Ti-U fluorescence microscope (Nikon) and the percentage of fluorescent cells was assayed by scoring 200 cells/field, three times and normalized to Hoechst staining.

### RNA extraction and semi-quantitative reverse transcription (RT)-PCR analysis

Cells and glioblastoma tissues were harvested in TRIzol Reagent (Invitrogen) and total RNA was isolated following the manufacturer’s instructions essentially as described [20]. PCR was performed by using genes specific oligonucleotides under conditions of linear amplification. PCR products were run on a 2% agarose gel and visualized by ethidium bromide staining using UV light. The housekeeping β-actin mRNA was used as internal control.

### Chromatin immunoprecipitation (ChIP) assay

Chromatin Immunoprecipitation (ChIP) analysis was carried out essentially as described [21]. Protein complexes were cross-linked to DNA in living cells by adding formaldehyde directly to the cell culture medium at 1% final concentration. Chromatin extracts containing DNA fragments with an average size of 500 bp were incubated overnight at 4°C with milk shaking using polyclonal anti-p53 antibody (FL393, Santa Cruz Biotechnology) and affinity purified rabbit anti-p73 antibody A300-126A (lot A300-126A-2, Bethyl Laboratories, Inc). Before use, protein G (Pierce) was blocked with 1 μg/μL sheared herring sperm DNA and 1 μg/μL BSA for 3 h at 4°C and then incubated with chromatin and antibodies for 2 h at 4°C. PCR was performed with HOT-MASTER Taq (Eppendorf) using 2 μL of immuniprecipitated DNA and promoter-specific primers for human *p21Waf1*, *Puma*, *p53AIP1*, *MDM2*, *MDR1*, and *cyclin B2* promoters. Immunoprecipitation with non-specific immunoglobulins (IgG; Santa Cruz Biotechnology) was performed as negative controls. The amount of precipitated chromatin measured in each PCR was normalized with the amount of chromatin present in the input of each immunoprecipitation. PCR products were run on a 2% agarose gel and visualized by ethidium bromide staining using UV light.

### Immunofluorescence of glioblastoma tissues

Human glioblastoma U373 cells were stably transfected with a pcDNA3-LUC vector using the cationic polymer LipofectaminePlus method, according to the manufacturer’s instructions (Invitrogen), as previously reported for *in vivo* imaging [22]. Mixed population were selected and luciferase activity was assayed on whole cell extract, compared to Mock cells (data not shown). Six-week-old CD-1 athymic nude (nu/nu) mice (Charles River Laboratories) were used for *in vivo* studies. All mouse procedures were carried out in accordance with Institutional standard guidelines. 2.5x10^5^ viable U373MG-LUC cells were inoculated into the brain of athymic nude mice and allowed to develop for about 6 days, as monitored by *in vivo* imaging (data not shown). For *in vivo* bioluminescence analysis, luciferase activity was quantified by IVIS Imaging System 200 (Caliper Life Sciences, Hopkinton, MA), as previously reported [22]. Mice were anesthetized with a combination (i.m., 2 mg/kg) of tiletamine-zolazepam (Telazol, Virbac, Carros, France) and xylazine (Xilazyne/Rompun, Bayer, Leverkusen, Germany) given i.m. at 2 mg/Kg. Then mice were injected i.p. with 150 mg/kg D-luciferin (Caliper Life Sciences) and imaged 10 to 15 minutes after injection. Data were acquired and analyzed using Living Image software version 3.0 (Caliper Life Sciences). After 6 days, mice were randomized in two groups (8 mice/group): 1) Mock-treated or 2) treated with Zn-curc (10 mg zinc/kg body weight), administrated every day by oral administration, over the course of one week. Glioblastomas were then harvested and stored in liquid nitrogen. Frozen tissue sections were analysed by a Nikon Eclipse Ti-U fluorescence microscope (Nikon) and the percentage of fluorescent cells was assayed by scoring 200 cells/field, three times and normalized to Hoechst staining.

### Statistics

All experiment unless indicated were performed at least three times. All experimental results were expressed as the arithmetic mean and standard deviation (s.d.) of measurements was shown. Student’s *t*-test was used for statistical significance of the differences between treatment groups. Statistical analysis was performed using analysis of variance at 5% (p < 0.05) or 1% (p < 0.01).

## Results

### Zn-curc complex induces apoptotic cell death in cancer cell lines carrying mtp53 (H175 and H273)

**Figure 1:**
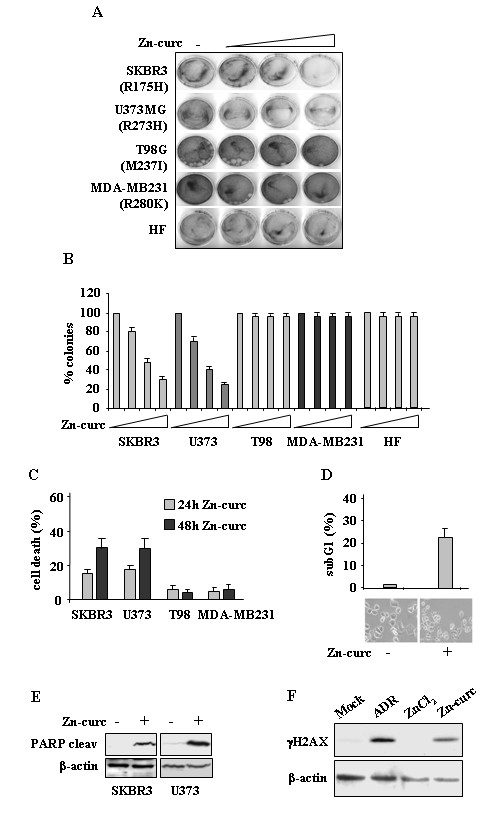
**Zn-curc impairs survival of mutant p53-carrying cells. (A)** Tumor cells (4 x 10^4^) were plated in 60 mm dish and 24 h later treated with increased amount of Zn-curc (20, 50, 100 μM). Twenty-four hours later, plates were washed with PBS and fresh medium was added. Death-resistant colonies were stained with crystal violet 14 days later. **(B)** Death-resistant colonies as in **(A)** were counted and plotted as percentage ± SD of two independent experiments performed in duplicate. **(C)** Cells (3 x 10^5^) were plated at subconfluence in 60 mm dish and the day after treated with Zn-curc for 24 and 48 h. Cell viability was measured by trypan blue exclusion assay and expressed as percentage ± SD of two independent experiments. **(D)** Cytofluorimetric analysis of the SubG1 peak evaluated by Propidium Iodide (PI) staining (upper panel) and microscopical analysis of SKBR3 cells, mock-treated or treated with Zn-curc (100 μM) for 24 h (lower panel). Percentage of apoptotic cells is shown ± SD of two independent experiments. **(E)** SKBR3 and U373 cells were treated with Zn-curc (100 μM) for 24 h. Equal amount of total cell extracts were subjected to immunoblot with anti-PARP (cleaved form, 87 Kd) or anti-β-actin antibodies. **(F)** RKO cells were treated with Zn-curc (100 μM), ZnCl_2_ (100 μM) or adryamicin (ADR, 2 μg/ml) for 24 h. Equal amount of total cell extracts were subjected to immunoblot with anti-γH2AX (phopho-Ser139) or anti-β-actin antibodies.

To evaluate the biological effect of Zn-curc complex we performed long-term survival assay in cancer cells lines carrying different p53 point mutations. Increasing doses of Zn-curc (20, 50, 100 μM) accordingly inhibited cell growth of SKBR3 (R175H) and U373 (R273H) cell lines while did not affect T98G (M237I) and MDA-MB231 (R280K) cell growth (Figure 1A), as evidenced by the quantification of the colony assays (Figure 1B). In our hands, Zn-curc did not affect long-term survival of normal human fibroblast (HF) (Figure 1A, 1B). Viability assay show that Zn-curc treatment induced time-dependent cell death only in SKBR3 and U373 cells compared to T98G and MDA-MB231 cells that were not affected (Figure 1C). Moreover, FACS analysis of SKBR3 cells stained with propidium iodide (PI) showed increased subG1 population after Zn-curc treatment, highlighting cell death (Figure 1D), as also evidenced by microscopic analysis (Figure 1D, lower panel). In agreement, the apoptotic marker PARP was cleaved in both SKBR3 and U373 cells after zinc treatment (Figure 1E). Finally, because Zn-curc has been reported to have DNA intercalating ability [13] we analysed the potential DNA damage occurring after treatment. As shown in Figure 1F, Zn-curc induced H2AX phosphorylation (γH2AX); as positive control of DNA damage we used the chemotherapeutic agent adryamicin (ADR) and as negative control we used ZnCl_2_ treatment. Together, these results suggest that Zn-curc exerted antiproliferative/apoptotic effects in mtp53-carrying cell lines, in particular with H175 and H273 mutations.

### Zinc-curc reactivates p53-DNA binding and transactivation activities

**Figure 2:**
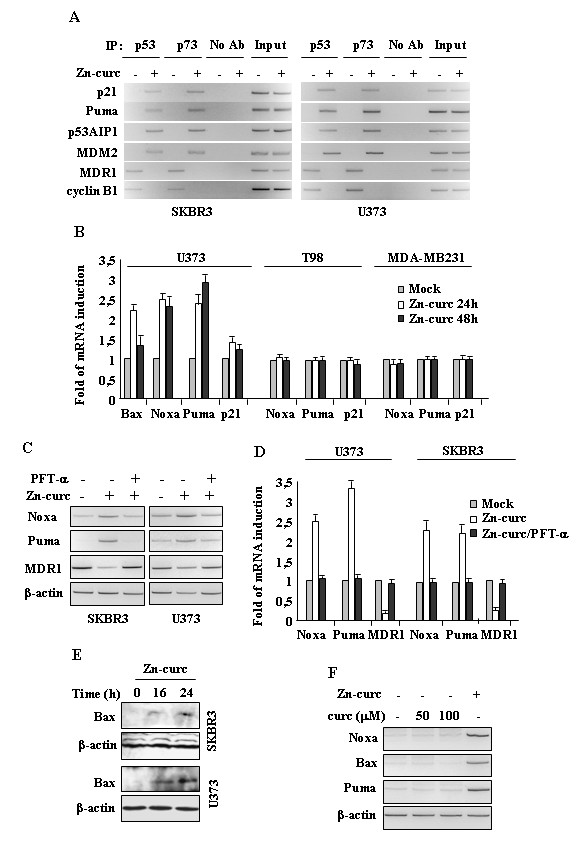
**Zn-curc restores wild-type p53-DNA binding and transactivating activities. (A)** SKBR3 and U373 cells (6x10^6^) were plated in 150 mm dish and the day after treated with Zn-curc (100 μM) for 16 h before assayed for chromatin immunoprecipitation analysis (ChIP) with anti-p53 or anti-p73 antibodies. PCR analyses were performed on the immunoprecipitated DNA samples using primers specific for wtp53 target gene promoters (p21, Puma, p53AIP1, MDM2) or for mtp53 target promoters (MDR1, cyclin B2). A sample representing linear amplification of the total chromatin (Input) was included as control. Additional controls included immunoprecipitation performed with non-specific immunogloblulins (No Ab). **(B)** Cells (3x10^5^) were plated at subconfluence in 60 mm dish and the day after treated with Zn-curc for 24/48 h. p53 target genes were detected by RT-PCR analysis. Gene expression was measured by densitometry and plotted as fold of mRNA expression over control (Mock), normalized to β-actin levels, ±SD. **(C)** SKBR3 and U373 cells were plated at subconfluence in 60 mm dish and the day after treated with Zn-curc (100 μM) for 24 h, with or without p53 inhibitor pifithrin-α (PFT-α) (30 μM). p53 target genes were dtected by RT-PCR analysis. β-actin was used as control. **(D)** Gene expression as in **(C)**, was measured by densitometry and plotted as fold of mRNA expression over control (Mock), normalized to β-actin levels, ±SD. **(E)** SKBR3 and U373 cells were treated with Zn-curc (100 μM) for the indicated hours and total cell extracts were subjected to immunoblot analysis. **(F)** U373 cells were plated at subconfluence in 60 mm dish and the day after treated with curcumin (Curc) (50, 100 μM) for 24 h. Zn-curc (100 μM for 24 h) was used as control of p53 activation. p53 target genes were detected by RT-PCR. β-actin was used as control.

To determine if the cell death and DNA damage induced by Zn-curc were correlated to reactivation of wild-type p53 activity, we performed chromatin immunoprecipitation (ChIP) analyses. The results revealed the ability of Zn-curc to restore p53-DNA binding activity to wild-type target gene promoters, including *p21*, *PUMA*, *p53AIP1*, and *MDM2*, to the detriment of mtp53-activated promoters, such as *MDR1* and *cyclin B1*[23,24] (Figure 2A). We also performed ChIP analyses using the p73 antibody because one of the mtp53 oncogenic characteristics is binding of the family member p73 with inactivation of p73 pro-apoptotic function [24,25]. Parallel to p53 results, ChIP analyses revealed that the p73 recruitment onto target promoters was induced after Zn-curc treatment, mirroring that of reactivated mt/wtp53 (Figure 2A). These results corroborate the findings that mtp53 can control molecules such as cyclin B1 and p73 that regulate, respectively, cell cycle progression and apoptosis, supporting its pro-tumorigenic effect.

We next compared the mRNA levels of p53 target genes (i.e., Bax, Noxa, Puma, p21) and found that Zn-curc increased the levels of all four p53 target genes analysed in U373 cells, particularly the apoptotic ones, while did not induce p53 target genes in T98G and MD-MB231 cells (Figure 2B). The specific effect of Zn-curc in reactivating p53 transactivation function was evaluated by using the p53 inhibitor pifithrin-α (PFT-α) [26] that indeed impaired the increase of wtp53 target genes in SKBR3 and U373 cells after Zn-curc treatment (Figure 2C), as confirmed by densitometric analyses (Figure 2D). Finally, immune-blot experiments show that Zn-curc treatment enhanced Bax protein levels in both SKBR3 and U373 cells (Figure 2E). These results support the findings that Zn-curc treatment was indeed restoring wtp53 transcriptional activity. As Zn-cur complex previously showed increased biological activity compared to curcumin alone [13,14], here we tested the effect of curcumin (curc) on p53 reactivation. We found that curcumin alone did not induce wtp53 target gene transcription (Figure 2F), suggesting that the effect of Zn-curc on mtp53 reactivation was mainly depended on Zn(II) ability to induce mtp53 reactivation.

### Zinc-curc induces conformational changes in p53-R175H and –R273H mutant proteins

**Figure 3:**
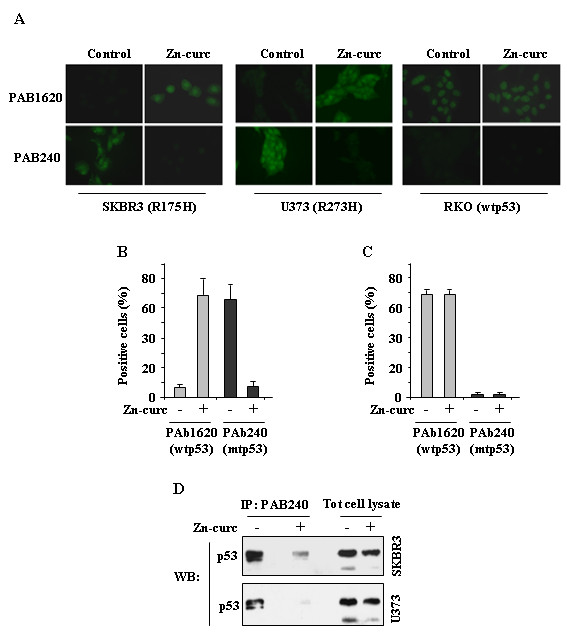
**Zn-curc induces a wild-type-like conformational change in mutant p53 proteins. (A)** Immunofluorescence of SKBR3 (H175) and U373 (H273) cells using p53-conformation-specific antibodies (PAB1620 for wt, folded conformation and PAB240 for mutant, unfolded conformation). Cells were treated with Zn-curc (100 μM) for 24 h before fixing and staining with antibodies. The RKO (wtp53) cell line is used as a control to show that the wtp53 conformation is not changed by Zn-curc treatment. Quantification of SKBR3 **(B)** or RKO **(C)** positive cells to PAB1620 and PAB240 antibodies before and after Zn-curc treatment, ±SD. **(D)** SKBR3 and U373 cells were treated with Zn-curc (100 μM) for 24 h. Total cell extracts were imunoprecipitated (IP) with conformation-specific antibodies (PAB1620 and PAB240) and then imunoblotted (WB) with anti-p53 (DO1) antibody. Input represents 1/10 of total cell extracts used for IP.

Because Zn-curc reactivated p53 transactivation function, we next analysed mtp53 protein conformation. Using immunofluorescence analyses we found that Zn-curc induced a conformation change in the R175H and R273H mutant p53 proteins that was recognized by the wild-type-specific antibody PAb1620 to detriment of the mutant-specific conformation detected by the antibody PAb240 (Figure 3A). Quantification of the fluorescence positive cells showed a strong reduction of PAb240 intensity whereas PAb1620 intensity was highly increased following Zn-curc treatment (Figure 3B). The RKO cell line, carrying wild-type p53 was used as a control to show that the wtp53 conformation was not changed by Zn-curc treatment (Figure 3A), as also shown by quantification analyses of fluorescent positive cells (Figure 3C). Immunoprecipitation analysis revealed that the p53 immunoreactivity to the PAb240 antibody remarkably reduced after Zn-curc treatment (Figure 3D). Altogether, these results demonstrate that Zn-curc modified the equilibrium between p53 mutant and wild-type conformation toward wild-type conformation.

### Zinc-curc localizes in glioblastoma tissues of an orthotopic mice model

**Figure 4:**
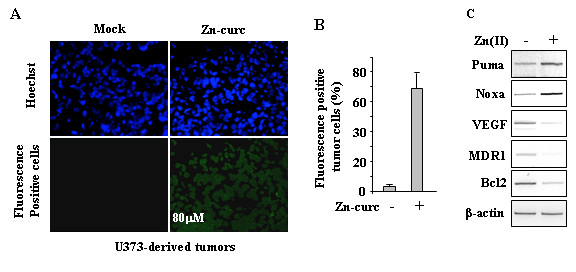
**Zn-curc reactivates mtp53 in an orthotopic U373 glioblastoma model. (A)** U373MG-LUC cells (2.5x10^5^) were injected into the brain of athymic mice and left to growth for 6 days before treating animals with Zn-curc every day for 7 days. Mock- or Zn-curc-treated U373M-derived tumors were then harvested and analysed with a fluorescent microscope that showed as diffuse fluorescence only in Zn-curc**-**treated tumors. **(B)** Quantification of tumor cell fluorescence positivity in U373-derived tumors, untreated or Zn-curc-treated, ±SD. **(C)** Total mRNA was extracted from harvested U373-derived tumors, untreated or Zn-curc-treated, and p53 target gene expression as well as VEGF, MDR1 and Bcl2 expression were assayed by PCR of reverse-transcribed cDNA. Gene expression was measured by densitometry and plotted as fold of mRNA expression over control (Mock), normalized to β-actin levels, ±SD.

Targeting a tumor tissue with a systemically administrated anticancer drug is of great importance especially for those tumors difficult to reach such as brain tumor where the blood-tumor barrier (BTB) plays a negative role. Therefore, we took advantage of the fluorescent feature of the Zn-curc compound [13,14] to evaluate its intratumoral localization. To this aim we used human U373 glioblastoma cells engineered with luciferase reporter (U373-LUC) for imaging analysis [22]. U373-LUC cells were injected into the brain of athymic nude mice. Ortothopic tumors were let to growth for 6 days, as evaluated by imaging analysis (data not shown), before treating animals with Zn-curc (10 mg/Kg) every day for 7 days. Glioblastoma untreated or treated tissues were then harvested and analysed with a fluorescent microscope that revealed a diffuse fluorescence into the glioblastoma tissues treated with Zn-curc, compared to the Mock-treated tumors (Figure 4A), as also evidenced by quantification of the fluorescence positive cells (Figure 4B). In addition, RT-PCR analyses of the U373-derived tumors showed reactivation of the wtp53 target genes (Puma and Noxa) only after Zn-curc treatment to detriment of mutant p53 target gene MDR1 (Figure 4C); moreover, VEGF and Bcl2 mRNA levels were markedly downregulated in the Zn-curc-treated tumors (Figure 4C). These findings indicate that Zn-curc complex can reach the intratumoral localization and modify molecular pathways for antitumor purpose.

## Discussion

Mtp53 proteins may drive tumor progression, metastasis and resistance to therapies [8]. In the clinic, the functional status of p53 has been associated with the prognosis, progression, and therapeutic response of tumors [27]. As a matter of fact, abrogation of mtp53 expression reduces tumor malignancy [28] and tumors containing wild-type p53 are usually more sensitive to radiotherapy or chemotherapy than those bearing mtp53 [29]. Moreover, earlier studies showed that the reconstitution of p53 has different biologic effects in tumor cells and in nontransformed cells [30,31]. Therefore, p53 reactivation is a promising anticancer strategy [32].

In the last years, many several small molecules have been claimed to reactivate mutant p53 by acting on the equilibrium of native and denatured protein, on the misfolded states, or by alleviating the mtp53 pro-oncogenic affects (i.e., mtp53/p73 interaction) [5,8]. We previously reported that the natural molecule ZnCl_2_ reverts p53 misfolding, thereby abrogating mtp53 pro-oncogenic function and increasing the response of mutant p53 tumor cells to anticancer drugs [9-12]. Zinc is a component of more than 3000 zinc-associated transcription factors, including DNA-binding proteins such as p53 [33]. Interestingly, p53 mutations are prone to loss of Zn(II) ion, which as a result promotes aggregation and therefore protein misfolding [4]. Many tumor-associated p53 mutations, classified as contact (e.g., R273H and R273C) or structural mutations (e.g., R175H, V143A, Y220C, G245S, R249S, F270L, R282W), may change the DBD conformation resulting in diminished DNA binding activity [34]. Zinc stabilizes the p53 DBD and is needed for wtp53 function [4], however, why in our hands ZnCl_2_ may influence specifically only R175H and R273H mutant proteins needs in-depth analysis.

The beneficial effects of ZnCl_2_ treatment as antitumor agent were shown in pivotal studies where zinc alone was reported to reduce tumor growth and aggressiveness with limited biotoxicity for instance in prostate cancer [35]. Very few studies, however, report the use of zinc in combination with chemotherapy, in fact as far as we know, zinc is not administered as part of any modern chemotherapy program in the treatment of cancer. Our previous pre-clinical studies performed in xenograft tumors show that ZnCl_2_ improves the chemotherapeutic effect reducing tumor growth compared to drug treatment alone [21]. This outcome could be reached because the ZnCl_2_ ability to target intratumoral hypoxia and restore p53 activity [21,22,36]. Moreover, *in vivo* studies in a mice tumor model with the transgenic MMTV-*neu* spontaneous breast cancer that develops p53 misfolding corroborated the findings that ZnCl_2_ reactivates misfolded p53 proteins and enhances antitumor effects of chemotherapy [12]. Interestingly, we recently demonstrated that zinc supplementation is required for the drug-induced immunogenic cell death in chemoresistant p53-functionally defective cancer cells [37] centering the 2 ideal goals of anticancer therapy that are the induction of a strong cytotoxic response of tumor cells [38] and the stimulation of host tumor-specific response, cooperating in the achievement of clinically relevant effects [39]. Altogether, these findings emphasize the translational potential of zinc in clinical practice.

Here we attempted to evaluate the effect of a novel Zinc(II) compound containing a 4,4′-disubstituted-2,2′-bipyridine as main ligand and curcumin and chloride as ancillary ligands [13,14]. As for ZnCl_2_, Zn-curc modified the equilibrium between p53 mutant and wild-type conformation toward wild-type conformation, specifically affecting R175H and R273H mutant proteins. Differently from ZnCl_2_ of our previous studies though [9-12], Zn-curc was able to directly induce apoptotic cell death likely due to p53 reactivation following both conformational changes and DNA damage induction, as evidenced by phosphorylation of histone γH2AX. Thus, Zn-curc metal complex combines DNA intercalating ability and cytotoxic activity with fluorescence [13,14]. This latter characteristic was in addition particularly useful in testing the capacity of Zn-curc to reach the tumor site *in vivo*. To this purpose, we used the ortothopic mice model of glioblastoma whose treatment remains a challenge due to its location, aggressive biological behaviour, angiogenesis and diffuse infiltrative growth, other than to the existence of blood-tumor barrier (BTB) representing an obstacle to the therapeutic efficacy via systemic administration [16,40]. Zn-curc was detected in the glioblastoma tissues, highlighting its capacity to reach the tumor site and affect molecular pathways important for tumor angiogenesis, and impairment of response to therapies such as VEGF, MDR1 and Bcl2. Targeting of such pathways might be important for restoring the response to anticancer therapies [41].

In summary, in this study we described the antitumor effect of a novel compound which combines the Zn(II) ability to reactivate some tumor specific p53 mutations with cytotoxic activity (due to its DNA intercalating ability) and fluorescence feature (due to the curcumin moiety). This Zn-curc complex might be useful in developing efficient anticancer drugs becuase (*i*) its ability to target one of the most common p53 mis-sense mutant, that is R1775H (http://www-p53.iarc.fr), (*ii*) its cytotoxic effect specific for tumor cells, and (*iii*) its capacity to cross the BTB when systematically administered.

## Abbreviations

BTB: Blood-tumor barrier; ChIP: Chromatin-immunoprecipitation; Curc: Curcumin; DBD: DNA-binding domain; mtp53: Mutant p53; PFT-α: Pifithryn-α; RT-PCR: Reverse-transcribed polymerase chain reaction; wtp53: Wild-type p53.

## Competing interests

The authors declare no competing financial interests.

## Authors’ contributions

AG carried out the ChIP, RT-PCR, and IP-WB studies, DT carried out the FACS studies; MP, CL and AS carried out the in vivo experiments and in vivo fluorescence studies; VDO participated in immunofluorescence studies; AC and DP supplied the Zn-curc reagent; MLA participated in the interpretation of the data; GDO conceived the experiments and wrote the paper. All authors read and approved the final manuscript.
